# Validation study of HSCL-10, HSCL-6, WHO-5 and 3-key questions in 14–16 year ethnic minority adolescents

**DOI:** 10.1186/s12875-016-0405-3

**Published:** 2016-01-27

**Authors:** Manjit Kaur Sirpal, Wench Haugen, Kaj Sparle, Ole Rikard Haavet

**Affiliations:** Department of General Practice, University of Oslo, Pb. 1130 - Blindern, N-0318 Oslo, Norway; Research Unit for General Practice, University of Aarhus, Aarhus, Denmark

**Keywords:** Adolescent, General practice, Depression, Diagnostic instrument

## Abstract

**Background:**

There is a lack of validated instruments for detection of depression in ethnic minority adolescent patients in primary care. This study aimed to compare a subgroup of the bilingual, ethnic minority adolescents with the rest of the population using Hscl-10, Hscl-6, WHO-5 and 3-Key Questions for detection of depression in primary care.

**Method:**

This is a cross-sectional, multicenter study conducted in General Practice in Norway and Denmark. A minor bilingual non-aggregated heterogenic ethnic minority group from non-European countries was compared with a major ethnic group of Norwegian/Danish adolescents. Participants completed questionnaires which were either mailed to them or found on our website. The Composite International Diagnostic Interview was used as gold standard. Depression classified by the International Classification of Diseases - 10. The Internal and external validity of the four questionnaires were examined. Optimal cut-off point for major depressive disorder was calculated using the Youden Index.

**Results:**

294 (77 %) were interviewed; mean age was 15 years. The ethnic group comprised 44 (64 % girls and 36 % boys). Chronbach’s alpha was above 0. 70 and area under curve was 0.80 or above for all instruments in the ethnic minority group. Cut-off points for major depressive disorder had sensitivities of 81 % (Hscl-10), 82 % (Hscl-6), 91 % (Who-5) and 81 % (3-key questions) in the ethnic minority group. Corresponding specificities were 80 % (Hscl-10), 77 % (Hscl-6), 80 % (Who-5) and 67 % (3-key questions). Cut-off points were the same Hscl-10, Who-5, the 3-key questions but differed for Hscl-6.

**Conclusion:**

Hscl-10, Hscl-6, WHO-5 and 3-key questions seem to be valid instruments for detection of depression in bilingual, ethnic minority adolescents in primary care.

## Background

There is a lack of validated instruments for detecting depression in ethnic minority adolescents in primary health care. Primary care physicians are the first line of contact with the health care system in Scandinavian and most other European countries [1, 2]. Minority groups often seek help for mental illness from primary care physicians [3, 4] over specialist mental health services [5], because they find that choice less stigmatizing [6]. However, Borowsky et al. [7] notes that primary care physicians are less likely to identify depression in various ethnic minority groups.

Primary care phenomenology differs considerably from specialist mental health: problems are presented in the form of unfiltered and unrecognized symptoms, not readily identifiable as mental health illness [8]. Symptom presentations in clinical settings depend upon subjective perception of somatic and psychological symptoms, which seem to be closely linked to ethnic background [8]. Asian adolescents demonstrate a tendency to somatize depressive symptoms [6] and African-American adolescents to express depressive symptoms as anger, aggression or irritability rather than hopelessness, sadness or depressive mood [9, 10] – expressions often misinterpreted and diagnosed as problem behaviour [11]. Similarly, Hispanic Americans use a variety of expressions to describe emotional distress: bad in the brain or in the mind, spiritually weak, craziness (locura), and losing control of oneself (perdiendo) [12]. Undoubtedly these culture bound expressions present a considerable challenge in diagnosis of depression.

Black adolescents are less likely to receive depression treatment than are their white counterparts [13] and ethnic minority adolescents living in western countries are at higher risk for depression [14–16] than are their majority peers. It is therefore crucial to improve diagnosis of depression in ethnic adolescents, as this condition is effectively treatable in this group [17, 18]. Detection of depression is improved by appropriate use of symptom rating scales in primary care in adolescent patients [19]. The National Institute for Health and Clinical Excellence (NICE; www.nice.org.uk/pdf/CG023quickrefguide.pdf) guidelines recommend the use of short questionnaires in primary care, for adults and adolescents.

Short questionnaires are less time consuming to use in primary care, but there is a need to validate diagnostic tools which can also be used for ethnic minority adolescents. Four short symptom rating scales varying in length – Hopkins symptom check list (Hscl-10) [20], Hopkins symptom check list (Hscl-6) (Sparle Christensen et al. 2015, personal communication), Well-being index WHO-5, and 3-key questions [21] (Haugen et al. 2015, personal communication) for young people aged 14–16 years living in Norway (Oslo) and Denmark (Aarhus) – have been validated in primary health care for Norwegian and Danish adolescents, Table 1.

**Table 1:** The four instruments validated in the study

Hscl-10	Hscl-6	Who-5	Key questions
Sudden fear for no reason	Feeling depressed	I have felt cheerful and in good spirits	During the past month you often been bothered by feeling down, depressed or hopeless?
Afraid & anxious	Feeling little worth	I have felt calm and relaxed	During the past month have you often been bothered by little interest or pleasure in doing things?
Faint & dizzy	Thoughts of taking your life	I have felt active and vigorous	Is this something you would like help with?
Tense & harassed	Feeling of being trapped	I woke up feeling fresh and rested	
Guilty	Feeling of loneliness	My daily life has been filed with things that interest me	
Sleeplessness	Feeling guilty		
Dejected			
Useless, of little worth			
Everything is a burden			
Hopelessness for the future			

The aim of this paper was to compare the subgroup of the bilingual, ethnic minority adolescents with the rest of the population using Hscl-10, Hscl-6, WHO-5 and 3-Key Questions for detection of depression in primary care.

## Methods

This study represents collaboration between the research Unit for General Practice, University of Aarhus, Denmark and the Department of General Practice, University of Oslo, Norway. In the first phase, GPs from the metropolitan area of Aarhus and Oslo were randomly recruited, and their 14–16 year old patients enrolled in the study. The GPs recorded their patients’ names, dates of birth and code numbers, which were used to identify participants and to serve as login codes for the web questionnaire. In the second phase, a letter of invitation with relevant information about the goal and procedure of the study was sent to patients, along with Hscl-10, Hscl-6, WHO-5 questionnaires (Table 1). In Norway, ethical standards required 14 and 15 year- olds and their parents to sign a consent form, included in the package. This was different in Denmark, where the ethical committee interpreted participation in the study as sufficient agreement to participate. Participating adolescents had the option of using the mail-in questionnaires or website, www.ungdep.au.dk, which was created for the study.

**Table 2:** Internal Validation of Hscl-10/6, who-5 and 3 key questions with the CIDI: Mokken scla e analysis and cronbach’s alpha for ethnic minority population

Instrument	Loevinger coefficient	Chronbach Alfa
HScl - 10	0,59	0,91
Hscl - 6	0,61	0,89
Who - 5	0,67	0,9
Key questions	0,77	0,74

Good scale: 1; Loevinger’s H > 0,50, ref Mokken RJ.A theory and procedure of scale analysis, 1971. 2; Chronbach’s alpha > 0,70,ref Bland JM, Altman DG. Statistics notes: Chronbach’s alpha. BMJ 1997;572

Responses were recognized by the login code and participation number. Participants were required to provide a telephone number by which they could be contacted (but not their names). This number was used to conduct The Composite International Diagnostic Interview (CIDI) by two members of the study group, who also conducted all CIDI interviews. The dates of receipt of the questionnaire and agreement form and of the telephone interview were recorded.

CIDI Version 2, 1 was used as the gold standard interview. CIDI is a widely accepted clinical instrument for measuring depression [22, 23], and also validated in ethnic minority populations [24]. A diagnosis within the last two weeks was defined as ongoing diagnosis. CIDI is a fully structured, standardized diagnostic interview that has been subjected to reliability and validity tests, and demonstrates good feasibility and high interrater reliability [25, 26]. The Demographic and Depression modules were used. In the CIDI interview we also registered if participants were mono- or bilingual and the second language was from Non-European countries (ethnic minority). The three key questions were asked before the CIDI interview.

Website data were collected directly in a data bank, and paper questionnaire and telephone interview date were also fed into the data bank. The data files were analysed by STATA version 11. The Danish Research Ethics Committee in Denmark (2006-2.0/44); and The National Committee for Research Ethics in Norway (S-06293a) approved this study.

### Statistical methods

Internal validity was examined using Cronbach’s alpha and the Mokken Scale Analysis, and Feldt’s W to test the equality of Cronbach’s alpha between the minor and the major ethnic population. The Mokken Scale was used to test that the questionnaires were unidimensional. External validity was analysed through Receiver Operating Characteristic curve (ROC), with specifying measurement for sensitivity, specificity and predictive values. Using the Youden Index optimal cut-off point for each instrument is decided, depicting the highest sensitivity and specificity values for the instruments validated. We used International Classification of Diseases 10 (ICD-10) diagnosis for mild depressive episode (F32.0), moderate depressive episode (F32.1), severe depressive episode without psychotic symptoms (F32.2), recurrent depressive disorder, current episode moderate (F33.1), recurrent depressive disorder, current episode severe without psychotic symptoms (F33.2) and Dysthymia (F34.1).

## Results

Mean age of the 2374 adolescents invited to participate was 15 years; 384 (16 %) returned the questionnaire), 294 (77 %) of which were interviewed. The ethnic minority group comprised 44 adolescents (64 % girls and 36 % boys). Questionnaires were returned an average of 23.5 days after receipt; 84 % of the ethnic minority group and 86 % of the majority group used the paper version. Among those interviewed on the CIDI, 25 % of ethnic adolescents and 11 % of majority adolescents met the ICD-10 criteria for major depression. All ethnic minority adolescents who completed CIDI also answered all three questionnaires and the 3-key questions.

### Internal validation

Internal validation was assessed by calculation with both Loevingers’s H (Table 2) and Cronbach’s alpha (Table 2). Loevinger’s H computes the observed and expected errors for each pair of items between a given item and all other items of a scale among all possible pairs of items in a scale. Loevinger’s H > 0. 5 is interpreted as a strong scale. Chronbach’s alpha is used as a measure of thereliability of a psychometric instrument. It provides an expression of increased consistency; it increases as the correlation between items increases. A psychometric instrument is generally considered reliable if Cronbach’s alpha is > 0.7. Chronbach’s alpha was above 0. 7 (good consistency) for all the instruments, irrespective of questionnaire length (Table 2). The scales had strong scalability as measured by Mokken H Statistic (H = 0, 59–0, 77) (Table 2), and satisfied the Mokken criteria.

**Table 3:**
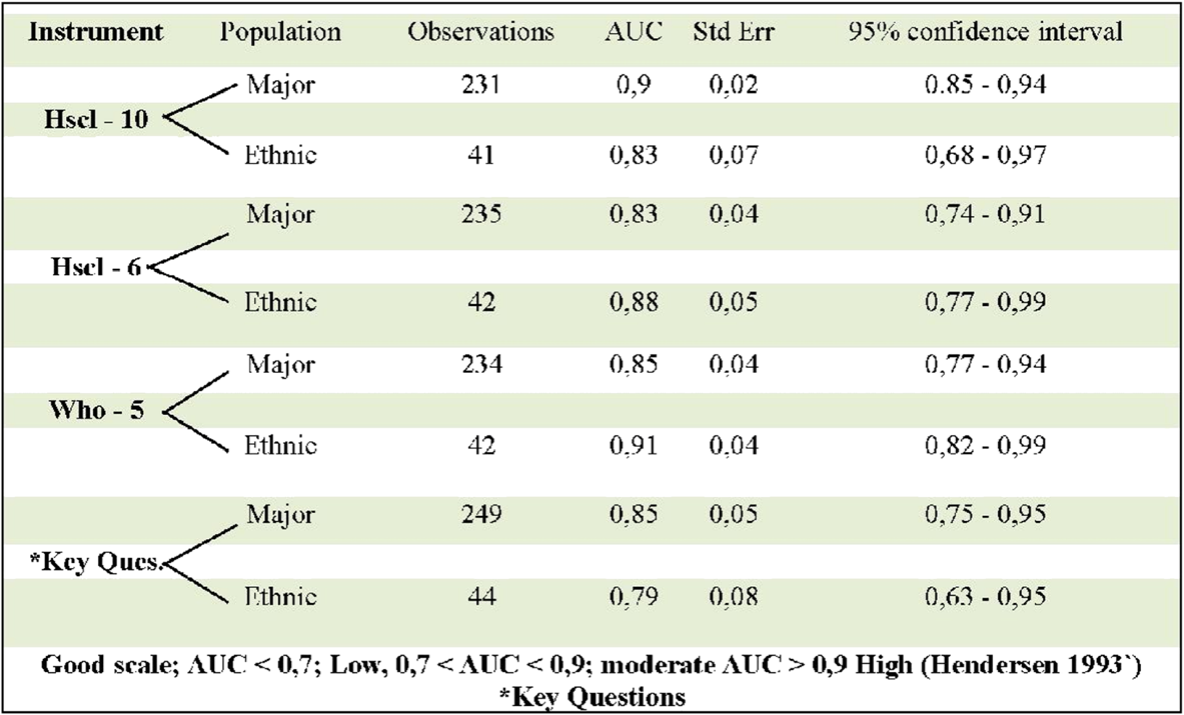
Comparison of external validety of HSc1-10, HSc1-6, who-5 & 3 key questions for major & ethnic population

Good scale; AUC < 0,7; Low < AUC < 0,9; moderate AUC > 0,9 High (Hendersen 1993)*Key Questions

### External validation

**Fig. 1:**
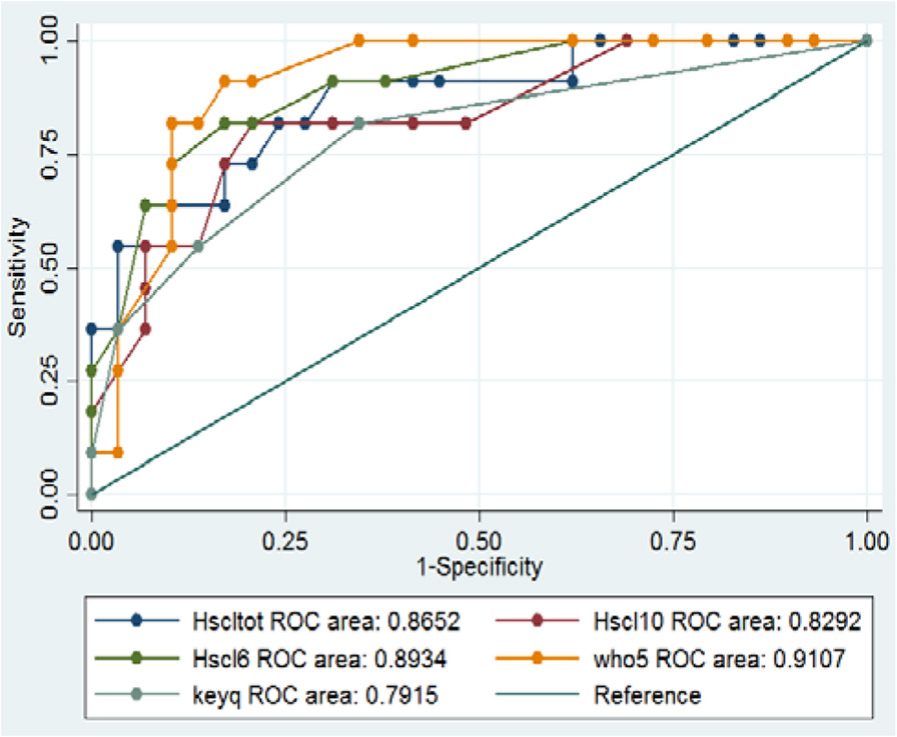
ROC Curves

External validity was examined by calculating ROC (Fig. 1). No significant differences in AUC (Area Under Curve) were observed between the majority and minority groups (Table 3) as the *p*-value is 0,19. No gender difference was found in the ethnic minority group.

### Cutoff point

The cutoff point for Hscl-10 was 16 for both ethnic and majority groups (sensitivity – ethnic 81 %, majority 90 %; specificity – ethnic 80 %, majority 79 %). Cutoff point for Hscl-6 was 11 for ethnic and 9 for majority (sensitivity – ethnic 82 %, majority 80 %; specificity – ethnic 77 %, majority 80 %). Cutoff point for Who-5 was 11 for both ethnic and majority (sensitivity – ethnic 91 %, majority 85 %; specificity – 80 % for both groups). For the three key questions, cutoff point was 1 for both groups (sensitivity – ethnic 81 %, majority 80 %; specificity – ethnic 67 %, majority 78. 5 %). The cutoff points were similar for girls and boys in the ethnic group.

## Discussion

For identification of depression in ethnic minority adolescents in primary care, 14–16 year olds in this study substantiate that Hscl-10, Hscl-6, WHO-5 and 3- key questions are all valid instruments. To our knowledge this is the first study to compare the validity of diagnostic instruments used for depression diagnosis in non-aggregated heterogenic ethnic minority adolescent population in primary care.

Our findings suggest equality in understanding and ability to relate to depression symptoms cross culturally in adolescents. Recent studies [27, 28] also report that the difference in expressing depression symptoms is less in bilingual adolescences cross culturally as compared to bilingual adults. As the cut-off points are the same for both genders, it makes questionnaires validated easily applicable in primary care. We found a higher percentage of adolescents with depression in the ethnic minority group than in the majority group. This finding is similar those of earlier studies [29] in emphasizing that ethnic minority adolescents may have a higher risk for depression [29].

These results provide primary care clinicians with the opportunity to use less time-consuming questionnaires to improve the diagnosis of depression in ethnic minority adolescents though it must be stressed that these instruments are merely an aid to clinical assessment [19]

This study has some limitations. First, the ethnic adolescent sub-group is small, so the results may differ concerning both validation and the cut-off points if studied in a larger population. However the method designed with the questionnaires are tested against a gold standard test (CIDI) on the same participant. It is a kind of individual test-retest method where the individual has a greater effect on the outcome than the sample. The area under the curve is not significantly different for the four questionnaires suggesting that the instruments show a positive tendency to be valid for use in the ethnic minority population studied. Second because the Questionnaires validated were not translated into the participants’ native languages, our results can only be applicable for bilingual adolescents.

We speculate that the ethnic minority adolescents in our study are highly acculturated. Third, the study did not include a clinical interview because the adolescents were interviewed by telephone; yet acceptable agreement between telephone and face-to-face assessment using an observer-rated depression scale has been found (30). CIDI is a fully structured interview that can be administered by trained lay interviewers [23], and these CIDI interviewers were two experienced GPs, trained and certified for CIDI.

## Conclusions

In summary, we conclude that Hscl-10, Hscl-6, Who-5 and the 3-key questions are valid instruments for detecting depression in ethnic minority adolescents from non-european countries in primary care when the language barrier is eliminated. Due to the small size of the ethnic minority population further research in larger populations is recommended.

The use of these short instruments together with diagnostic dialogue can help primary health clinicians to translate patients’ narrative into medically accepted terminology. The use of questionnaires will also enhance accurate diagnosis of depression in ethnic minority adolescents, leading to better treatment, better outcome and aids in communication with other health services. We recommend use of standardized validated instruments in future research studies concerning depression in ethnic minority adolescents.
